# Supplementary material for: WordCommentsAnalyzer: A windows software tool for qualitative research
Source: F1000Res. 2018 Sep 4;7:536. Originally published 2018 May 3. [Version 2] doi: 10.12688/f1000research.14819.2 (PMC6137411; doi:10.12688/f1000research.14819.2)
Supplement: Supplementary file 3 [file f1000research-7-17614-s0002.tgz › b2a79496-4ada-47c4-9682-a99ce867836d.zip!/Eight Iranian Health Journals/Archives of Rehabilitation_18.docx]

© Archives of Rehabilitation. This work is licensed under CC BY-NC 4.0 (https://creativecommons.org/licenses/by-nc/4.0/).

Volume & Issue: دوره 14 -

Title: هَماکارها؛ پنجره‌ای باز به دنیای مراقبت از کودکان فلج مغزی

http://rehabilitationj.uswr.ac.ir/article-1-1380-fa.html

هدف: هَماکار، به مشغولیت دو یا چند نفر در یک کاره اطلاق می‌گردد که افراد به‌صورت فعالانه و در یک چارچوب زمانی، به‌صورت جسمی، هیجانی و فکری در آن درگیر هستند. هدف از این مطالعه، تبیین هَماکارهای مادران کودکان فلج مغزی از دیدگاه مادران، کودکان فلج مغزی و کاردرمانگران در جامعه ایرانی بود. روش بررسی: این مطالعه کیفی قسمتی از یک مطالعه نظریه پایه است که با مشارکت ۳ کودک فلج مغزی، ۱۳نفر از مادران آنها و ۶ کاردرمانگر انجام شد. افراد با استفاده از روش نمونه‌گیری هدفمند و در ادامه نمونه‌گیری نظری انتخاب شدند. داده‌ها با استفاده از مصاحبه فردی و گروهی به‌صورت نیمه‌ساختارمند، مشاهده هدفمند در منزل و کلینیک، فیلم ویدئویی و خاطره جمع‌آوری شد. کلیه مصاحبه‌ها ضبط شده، بازنویسی و مرور شدند. سپس به روش تحلیل محتوی کیفی و با استفاده از آنالیز مقایسه مداوم مورد تجزیه و تحلیل قرار گرفتند. یافته‌ها: از مجموع مصاحبه‌ها، ۶۹۵ کد باز یا اولیه بدست آمد. این کدها در طی فرایند تجزیه و تحلیل در چهار درونمایه اصلی: مواجهه با مشکلات مراقبت فردی، پی‌جوئی برای درمان، چالش‌های تطابقی مراقبت آموزشی و محدود شدن اوقات فراغت والدین به عنوان هَماکارهای‌ اصلی مادران کودکان فلج مغزی طبقه‌بندی شدند.  نتیجه‌گیری: شناخت هَماکارها، نگرش ما را نسبت به مراقبت‌های ویژه‌ای که مادران کودکان فلج مغزی، علاوه بر مراقبت‌های معمولی، در قبال فرزندانشان انجام می‌دهند توسعه داده و به مدیریت مراقبتی کودک برای مدت زمان طولانی کمک کرده و آگاهی ما را از مشکلات و موانع مراقبتی افزیش می‌دهد.

Title: تدوین نسخه فارسی آزمون غربالگر سنجش تکامل کودکان «بِیلی» و تعیین اعتبار و روایی آن

http://rehabilitationj.uswr.ac.ir/article-1-1418-fa.html

هدف: این مطالعه به‌منظور تدوین نسخه فارسی روا و دارای اعتبار آزمون غربالگر سنجش تکامل کودکان بیلی انجام گرفت. روش بررسی: مراحل ترجمه، باز ترجمه، تعیین روایی صوری و محتوایی و تطبیق فرهنگی و زبانی برای پنج حیطه تکاملی آزمون به‌ترتیب انجام شد. سپس یک مطالعه پایلوت بر روی ۴۵ کودک فارسی زبان ۱ تا ۴۲ ماهه در تهران در سال ۱۳۹۱ اجرا گردید تا میزان وضوح عناوین، تطابق فرهنگی و تشخیص سوالات مبهم مشخص گردد. در ارزیابی اعتبار آزمون از سه روش برآورد همسانی درونی، بازآزمایی و اعتبار ارزیاب‌ها در ۳۴ کودک استفاده شد. سپس برای تعیین خصوصیات روان‌سنجی آزمون، تعداد ۲۶۰ کودک ۱ تا ۴۲ ماهه از مراکز سلامت و مراقبتی واقع در پنج منطقه شهر تهران، به‌صورت نمونه‌گیری مستمر انتخاب شدند و آزمون برای ایشان اجرا گردید. یافته‌ها: در مناسب‌سازی فرهنگی و زبانی که توسط تیم تخصصی انجام شد، برخی تغییرات غیرقابل اجتناب بود. در تمامی گروه‌های سنی در حیطه‌های ارتباطی تغییراتی داده شد که به‌طور عمده منشا زبانی یا فرهنگی داشتند. ضریب همسانی درونی در خرده‌آزمون‌های شناختی، ارتباط درکی و بیانی، حرکات ظریف و درشت به‌ترتیب ۰/۹۶، ۰/۹۵، ۰/۹۵ و ۰/۹۴ به‌دست آمد و روایی سازه آزمون با روش تحلیل عاملی و مقایسه نمره‌ها در گروه‌های سنی تایید شد. نتیجه‌گیری: نسخه فارسی آزمون غربالگر سنجش تکامل کودکان «بِیلی» دارای اعتبار و روایی است.

Title: اثربخشی بازآموزی اسنادی بر اضطراب دانش‌آموزان با ناتوانی‌های یادگیری

http://rehabilitationj.uswr.ac.ir/article-1-1325-fa.html

هدف: هدف از مطالعه حاضر، تعیین تأثیر برنامه گروهی بازآموزی اسنادی بر اضطراب دانش‌آموزان با ناتوانی‌های یادگیری شهر تهران بود. روش‌ بررسی: در این پژوهش شبه‌آزمایشی از طرح پیش‌آزمون و پس‌آزمون با گروه کنترل استفاده شد. از جامعه آماری دانش‌آموزان با ناتوانی‌های یادگیری شهر تهران در سال تحصیلی ۹۲-۱۳۹۱، ۲ مرکز اختلال یادگیری به روش هدفمند و در دسترس (۳۶ نفر؛ ۹ دختر و ۲۷ پسر پایه‌های دوم تا ششم ابتدایی و سنین ۷ تا ۱۲ سال) انتخاب شدند. دانش‌آموزان یک مرکز به‌طور تصادفی به‌عنوان گروه آزمایش و دانش‌آموزان مرکز دیگر به‌عنوان گروه کنترل (هر گروه ۱۸ نفر) در نظر گرفته شدند. گروه آزمایش به ۳ گروه فرعی ۶ نفری تقسیم شدند و هر گروه طی ۱۱ جلسه مداخله ۴۵ دقیقه‌ای در مدت ۶ هفته (دو روز در هفته) آموزش بازآموزی اسنادی گروهی را دریافت نمودند. در صورتی که گروه کنترل فقط در برنامه‌های متداول مرکز شرکت کردند. پرسشنامه اضطراب کودکان اسپنس قبل و بعد از مداخله توسط دو گروه آزمایش و کنترل تکمیل شد. داده‌ها با استفاده از آزمون t وابسته و تحلیل کوواریانس تجزیه و تحلیل شد. یافته‌ها: نتایج تحلیل کوواریانس نشان داد که مداخله بازآموزی اسنادی بر میزان اضطراب تأثیر نداشته‌است و بین اضطراب دو گروه آزمایش و کنترل تفاوت معنادار (P=0/34) وجود ندارد. نتیجه‌گیری: برنامه گروهی بازآموزی اسنادی احتمالاً قادر به کاهش نشانه‌های اضطراب در دانش‌آموزان با ناتوانی‌های یادگیری نیست.

Title: کارآمدی راهبرد مرور ذهنی بر حافظه کوتاه‌مدت شنیداری کودکان آموزش‌پذیر ۵تا۸ سال مبتلا به نشانگان داون

http://rehabilitationj.uswr.ac.ir/article-1-1383-fa.html

هدف: یکی از مشکلات کودکان مبتلا‌به نشانگان داون، عملکرد ضعیف آنها در نگهداری اطلاعات و یادآوری آنها از حافظه است. تحقیق حاضر به‌منظور تعیین کارآمدی راهبرد مرور ذهنی بر حافظه کوتاه‌مدت شنیداری کودکان آموزش‌پذیر ۵ تا ۸ سال مبتلا‌به نشانگان داون انجام شد. روش بررسی: در این پژوهش شبه‌آزمایشی، ۲۴ کودک (۱۴ پسر و ۱۰ دختر) مبتلا‌به نشانگان داون به‌روش در دسترس از کانون خیریه سندرم داون ایران انتخاب شدند و با استفاده از ماتریس‌های پیشرونده هوش ریون مورد ارزیابی قرار گرفتند. سپس کودکان به‌طور تصادفی در گروه‌های آزمایش و کنترل (هر گروه ۱۲ نفر) قرار داده شدند. گروه آزمایش در ۸ جلسه (هفته‌ای ۲ جلسه و هر جلسه ۳۰ دقیقه) آموزش گروهی راهبرد مرور ذهنی شرکت کردند. همه آزمودنی‌ها با استفاده از آزمون توالی حافظه شنیداری-بیانی قبل از شروع مداخله آموزشی و پس از آن مورد ارزیابی قرار گرفتند. داده‌ها با بهره‌گیری از روش تحلیل کوواریانس تک‌متغیری تحلیل شدند. یافته‌ها: نتایج حاکی از تحلیل کوواریانس بیانگر آن بود که راهبرد مرور ذهنی موجب افزایش فراخنای عدد، فراخنای کلمه و حافظه کوتاه‌مدت شنیداری گروه آزمایش در مقایسه با گروه کنترل شده‌است (P<0/01). نتیجه‌گیری: می‌‌توان نتیجه گرفت که آموزش راهبرد مرور ذهنی به‌عنوان روش مؤثر در بهبود فراخنای عدد، فراخنای کلمه و حافظه کوتاه‌مدت شنیداری در کودکان مبتلا‌به نشانگان داون به‌شمار می‌‌رود و دربرگیرنده تلویحات کاربردی برای آموزش آنها است.

Title: تاثیر آموزش پاسخ‌محور بر مشکلات رفتاری کودکان ۴تا۶ سال دارای اوتیسم

http://rehabilitationj.uswr.ac.ir/article-1-1395-fa.html

هدف: مطالعه حاضر با هدف تعیین اثربخشی آموزش پاسخ‌محور بر مشکلات رفتاری کودکان ۴ تا ۶ سال دارای اوتیسم انجام گرفت. روش بررسی: پژوهش حاضر یک مطالعه شبه‌آزمایشی با طرح سری‌های زمانی دو گروهی بود. ۱۲ نفر (۳ دختر و ۹ پسر) به شیوه تمام‌شماری از کودکان دارای اوتیسم مراجعه کننده به کلینیک ذهن زیبا شهر تهران در سال ۱۳۹۱ انتخاب شدند و به تعداد مساوی در دو گروه آزمایش و کنترل قرار گرفتند. گروه آزمایش طی ۴۵ روز (هفته‌ای ۳ جلسه، جلسه‌ای ۱ ساعت) فقط آموزش پاسخ‌محور را دریافت نمودند، در حالی که گروه کنترل تنها از روش‌های آموزشی و درمانی مدون کلینیک (تحلیل رفتار کاربردی و گفتاردرمانی) بهره‌مند شدند. تمامی کودکان با استفاده از مقیاس درجه بندی اوتیسم گیلیام (GARS) ۳ بار قبل از مداخله و ۳ بار پس از آن ارزیابی شدند. داده‌ها نیز با استفاده از تحلیل کوواریانس مورد تجزیه و تحلیل قرار گرفتند.  یافته‌ها: نتایج تحلیل کوواریانس نشان داد که نمره رفتارهای کلیشه‌ای و مهارت‌های ارتباطی در دو گروه آزمایش کاهش یافت، ولی از نظر آماری تفاوت معناداری بین گروه آزمایش وکنترل وجود نداشت (P=0/45). همچنین نتایج بیانگر بهبود معنی‌دار تعامل‌های اجتماعی گروه آزمایش در مقایسه با گروه کنترل بود (P<0/001). نتیجه‌گیری: می‌توان نتیجه گرفت که برنامه آموزش پاسخ‌محور بر تعامل‌های اجتماعی کودکان دارای اوتیسم تأثیر مثبت دارد، ولی این تأثیر در مورد رفتارهای کلیشه‌ای و مهارت‌های ارتباطی معنادار نبود.

Title: تأثیر آموزش تقلید متقابل بر مهارتهای اجتماعی کودکان دارای اوتیسم

http://rehabilitationj.uswr.ac.ir/article-1-1409-fa.html

هدف: پژوهش حاضر با هدف تعیین تاثیر آموزش تقلید متقابل بر مهارت های اجتماعی کودکان دارای اوتیسم انجام شد. روش بررسی: این مطالعه از نوع شبه آزمایش با طرح اندازه گیری های مکرر بود. از میان تمامی کودکان دارای اوتیسم با عملکرد بالا در سال تحصیلی 92-1391 با استفاده از نمونه گیری در دسترس و هدفمند، 14 کودک 5 تا 7 سال (3 دختر و 11 پسر) ارجاع داده شده به یک مرکز خصوصی انتخاب و به تعداد مساوی در دو گروه کنترل و آزمایش قرار گرفتند. کودکان با استفاده از پرسشنامه نیمرخ مهارت های اجتماعی اوتیسم (بلینی، 2006) 3 بار قبل از مداخله آموزشی و 3 بار بعد از آن، از لحاظ مهارتهای اجتماعی ارزیابی شدند. گروه آزمایش افزون بر خدمات آموزشی مرکز در 20 جلسه آموزش تقلید متقابل (هفته ای 2 جلسه یک ساعته، انفرادی) شرکت کردند ولی گروه کنترل در این جلسات حضور نیافتند. نتایج با استفاده از تحلیل واریانس چند متغیری با اندازه گیری های مکرر مورد تجزیه و تحلیل قرار گرفت. یافته‌ها: نتایج تحلیل واریانس با اندازه گیری های مکرر بیانگر آن بود که دریافت برنامه آموزش تقلید متقابل به طور کلی منجر به افزایش معنادار (0/027=P) مهارت‌های اجتماعی، تعامل ‌اجتماعی و مشارکت ‌اجتماعی کودکان دارای اوتیسم شده است. در حالی که رفتارهای زیانبخش اجتماعی به طور معنادار کاهش نیافته بودند (0/05>P). نتیجه‌گیری: می توان نتیجه گرفت که برنامه آموزش تقلید متقابل منجر به بهبود مهارت های اجتماعی، تعامل اجتماعی و مشارکت اجتماعی کودکان دارای اوتیسم می‌ شود و استفاده از این برنامه در محیط های آموزشی و اجتماعی کودکان با اوتیسم و نیز به عنوان بخشی از برنامه آموزش خانواده و یا یکی از محورهای اصلی آموزشی در طراحی برنامه های پیش دبستانی کودکان دارای اوتیسم پیشنهاد می شود.

Title: عوامل موثر بر تاخیر در مراجعه برای خدمات گفتاردرمانی در کودکان ایرانی با اختلالات گفتار و زبان

http://rehabilitationj.uswr.ac.ir/article-1-1428-fa.html

هدف: شناسایی زود هنگام کودکانی که در معرض خطر یا در مراحل ابتدایی تاخیر تکاملی گفتار و زبان خود قرار دارند برای ارایه خدمات مداخله به هنگام بسیار ضروری است. اما متاسفانه این اختلالات در کشور ما به موقع تشخیص داده نشده یا برای درمان و توانبخشی ارجاع داده نمی‌شوند. روش بررسی: فاز اول مطالعه به شناسایی عوامل بالقوه موثر بر تاخیر در مراجعه‌ی به هنگام در کودکان دارای اختلالات گفتار و زبان، از طریق جستجوی منابع و نیز از دیدگاه صاحب نظران و خانواده‌ها اختصاص داشت. در این مرحله ۱۲ نفر از صاحبنظران و ۹ نفر از والدین کودکان دارای اختلالات گفتار و زبان بود از طریق مصاحبه عمیق نیمه ساختار یافته فهرست اولیه عوامل تهیه شده بر اساس مرور منابع را تکمیل کردند. سپس بر آن اساس پیش نویس پرسشنامه عوامل تاخیر در مراجعه‌ تهیه و تنظیم گردید و از نظر روایی صوری و محتوایی و پایایی به تایید رسید. آنگاه پرسشنامه در اختیار دو گروه والدین کودکان دارای اختلالات گفتار و زبان که یک گروه بعد از 3 سالگی(30 نفر) و گروه دیگر پیش از ۳ سالگی (30 نفر) جهت گفتاردرمانی مراجعه کرده بودند، به عنوان گروه مورد و شاهد قرار گرفت. یافته‌ها: از میان هفت عاملی که قبل از به تشخیص قطعی رسیدن اختلال گفتار و زبان کودک بین دو گروه مورد و شاهد تفاوت معنادار نشان دادند، سه عامل به دریافت مشاوره و راهنمایی صحیح توسط پزشکان، دو عامل به آگاهی والدین و دو عامل دیگر به انجام غربالگری برای کودک ارتباط داشتند. هر شش عاملی که بعد از به تشخیص قطعی رسیدن اختلال گفتار و زبان کودک بین دو گروه مورد و شاهد تفاوت معنادار نشان دادند، به نحوی به باورها و نگرشهای ناصحیح والدین ارتباط داشته اند. نتیجه‌گیری: توصیه می شود اولا برنامه های آگاهسازی عمومی به منظور ارتقای آگاهی و بهبود باور و نگرش اقشار مختلف مردم تدارک دیده شود و دوما آموزشهای مناسبی برای ارتقای سطح دانش و بهبود نگرش متخصصان کودکان و سایر پزشکان مرتبط با کودکان در این زمینه به عمل آید.

Title: بررسی درد اسکلتی-عضلانی مزمن در مادران دارای کودکان مبتلا به فلج مغزی

http://rehabilitationj.uswr.ac.ir/article-1-1297-fa.html

هدف: اکثرکودکان فلج مغزی در زندگی خود نیازمند مراقبت های خاص و طولانی مدت می باشند که عمدتاً توسط مادران آن ها فراهم می گردد و فراهم کردن این نیازهای مراقبتی منجر به افزایش فشارهای جسمی به سیستم اسکلتی-عضلانی مادران آنها می گردد.  هدف از مطالعه حاضر بررسی درد های اسکلتی-عضلانی مزمن در مادران کودکان مبتلا به فلج مغزی می باشد. روش بررسی: این مطالعه توصیفی در سال 2011 در تهران انجام شد که در آن دو گروه مادران دارای کودکان 4 تا 12 ساله فلج مغزی و سالم که به روش نمونه گیری غیر احتمالی ساده به مطالعه وارد شدند. جهت ارزیابی درد اسکلتی-عضلانی از پرسشنامه نوردیک استفاده شد و از طریق آزمون تی مستقل و همبستگی پیرسون ارتباط میان متغیر ها بررسی شد. یافته ها: در این مطالعه 60 مادر دارای کودک فلج مغزی و 60 مادر دارای کودک سالم شرکت کردند که میانگین و انحراف معیار سن بر حسب سال در مادران 02/6±79/33 و در کودکان 71/2± 7/11 مشاهده شد. در مادران کودکان فلج مغزی افسردگی و درد اسکلتی عضلانی مزمن به طور معناداری بالاتر بود (P<0/05) و درد در ناحیه کمر، شانه، مچ و انگشتان پا نیز در این مادران به طور معناداری بالاتر مشاهده شد (P<0/05). نتیجه گیری: با توجه به افزایش تجربه درد اسکلتی-عضلانی مزمن در مادران دارای کودکان فلج مغزی جهت کاهش اثرات نامطلوب فعالیت های مراقبتی، این مادران نیازمند حمایت های جسمانی و روانی می باشند. پیشنهاد می گردد دوره های آموزش نحوه صحیح مراقبت از خود و کودک برای این مادران برگزار گردد تا مشکلات جسمی و روانی ناشی از مراقبت در این مادران کاهش یابد.

Title: مقایسه بلوغ هیجانی در پسران نوجوان با سندروم داون (تحقیقی در مدارس عادی و استثنایی)

http://rehabilitationj.uswr.ac.ir/article-1-1414-fa.html

هدف: این مطالعه به بررسی مقایسه ای بلوغ هیجانی و مشکلات مربوطه در افراد با سندروم داون پسر (18-12) ساله ای میپردازد که در مدارس عادی و استثنایی درس میخوانند . روش‌ بررسی: دو گروه ۱۵ کودک با سندروم داون ۱۲ تا ۱۸ ساله پسر که در مدارس عادی و استثنایی درس میخوانند با توجه به متغییرهای بهره هوشی, سن ,تحصیلات خانواده و وضعیت اجتماعی – اقتصادی همسان سازی گردیدند و جداگانه با استفاده از پرسشنامه مقیاس بلوغ هیجانی افراد مورد ارزیابی و بررسی قرار گرفتند. یافته‌ها: افراد با سندروم داون که در مدارس عادی درس میخوانند به طور معناداری (P<0/001) در مقیاسهای بلوغ هیجانی و مولفه های آن (ثبات و بازگشت هیجانی و نیز سازگاری اجتماعی و استقلال) اختلاف دارند. نتیجه‌گیری: مهارتهای اجتماعی در نوجوانان با سندروم داونی که در مدارس عادی تحصیل میکننددر مقایسه با همتایان خود که در مدارس استثنایی درس میخوانند رشد مناسب تری دارد. این تنیجه میتواند تاییدی در بسط آموزش فرا گیر باشد.

Title: مشکلات حرکتی دختران با نشانگان داون در مقایسه با دختران کم توان ذهنی در کودکان سنین مدرسه

http://rehabilitationj.uswr.ac.ir/article-1-1400-fa.html

هدف: عملکرد حرکتی کودکان با نشانگان داون مشابه کودکان کم توان ذهنی است، اما حرکاتشان کندتر بوده و کفایت ضعیف تری دارد. هدف این مطالعه، شناسایی نقاط ضعف در عملکرد حرکتی دختران با نشانگان داون با استفاده از تست حرکتی برونینکس اوزرتسکی می باشد. روش‌ بررسی: در این مطالعه توصیفی-تحلیلی-مقطعی، 36 کودک دختر کم توان ذهنی در سنین 8 تا 13 سال شرکت داشتند. 18 نفر از آنان مبتلا به نشانگان داون بوده، که با 18 نفر کم توان ذهنی مقایسه شدند. در گروه نشانگان داون، از نمونه های در دسترس استفاده شد. آزمودنی های گروه کم توان ذهنی به روش نمونه گیری تصادفی ساده انتخاب شدند. دو گروه شرکت کننده از نظر سطح هوشبهر و سن تقویمی همتاسازی شدند. ابزار مطالعه آزمون برونینکس اوزرتسکی بود. داده های حاصل با استفاه از آزمون ناپارامتری یوی من ویتنی و t-test تحلیل شد. یافته‌ها: عملکرد حرکتی گروه نشانگان داون در عملکرد حرکتی درشت (P<0/014) خرده آزمون های تعادل (P<0/029)، زمان واکنش (P<0/034) و کنترل بینایی حرکتی (P<0/048) به طور معناداری ضعیف تر از گروه کم توان ذهنی بود. ولی در عملکرد حرکتی ظریف و عملکرد حرکتی کلی و در خرده آزمون های سرعت و چالاکی دویدن، قدرت، هماهنگی اندام فوقانی، سرعت و دقت اندام فوقانی، تفاوت معناداری بین دو گروه مشاهده نشد. نتیجه‌گیری: افراد کم توان ذهنی به ویژه افرد با نشانگان داون به برنامه توانبخشی حرکتی در دوره نوجوانی نیاز دارند. کلید واژه ها: مهارت حرکتی/ کم توان ذهنی/ نشانگان داون/ تست حرکتی برونینکس اوزرتسکی

Title: بررسی تکامل حرکتی کودکان 1 تا 4 ساله فنیل کتونوری تحت رژیم درمانی از بدو تولد

http://rehabilitationj.uswr.ac.ir/article-1-1376-fa.html

هدف: بررسی مهارت های حرکتی درشت و ظریف کودکان 1 تا 4 ساله فنیل کتونوری تحت رژیم درمانی از بدو تولد می باشد. روش بررسی: این مطالعه به روش تحلیلی مقطعی، بر روی 70 کودک 1 تا 4 ساله فنیل کتونوری، از نمونه در دسترس بر اساس لیست بیماران کلینیک ارجاع فنیل‌کتونوری انجام شد. این کودکان توسط غربالگری از بدو تولد تشخیص داده شده و از بدو تولد تحت درمان بوده اند. 100 کودک نیز به عنوان گروه شاهد از مهد کودک‌های تهران به طور تصادفی انتخاب شدند. ابزار گردآوری اطلاعات پرسشنامه دموگرافیک، آزمون پی بادی و ارزیابی بالینی توسط متخصص کودکان بود. پس از جمع آوری داده ها ، تحلیل داده‌ها با استفاده از نرم‌افزار SPSS نسخه 19 انجام شد. یافته‌ها: متوسط سن کودکان 29/7 (±3/11) ماه در گروه شاهد و 28/5 (±6/11) ماه در گروه مورد بود. بین دو گروه از نظر نحوه زایمان، دور سر، قد و وزن فعلی و بدو تولد رابطه معناداری وجود نداشت. بین دو گروه از نظر تکامل مهارت‌های حرکتی درشت (P=0/010)، ظریف (P<0/001) و کلی (P<0/001) اختلاف معناداری وجود داشت . نتیجه‌گیری: نتایج این پژوهش نشان داد که مداخلات غربالگری و رژیم درمانی رایج کنونی در کودکان فنیل کتونوری، تاخیرهای حرکتی را به طور کامل برطرف نکرده و سطح تکامل حرکتی در کودکان مبتلا به فنیل کتونوری درمان شده با رژیم درمانی از بدو تولد کمتر از کودکان عادی است. لذا تشخیص و مداخله زودرس توانبخشی جهت کلیه کودکان درمان شده با رژیم درمانی از بدو تولد و در سنین پایین توصیه می‌شود.

Title: بررسی تاثیر تطبیق منزل بر سطح تکامل در کودکان فلج مغزی 15 تا 60 ماهه

http://rehabilitationj.uswr.ac.ir/article-1-1416-fa.html

هدف: هدف این مطالعه تعیین تاثیر تطبیق منزل بر سطح تکامل در کودکان فلج مغزی 15 تا 60 ماهه می باشد. روش بررسی: در این مطالعه ی تجربی، 40کودک 15 تا60 ماهه ی فلج مغزی از بین مراجعه کنندگان به کلینیک های توانبخشی تهران مطابق معیارهای تحقیق انتخاب شدند و پس از همتاسازی از نظر نوع فلج مغزی، نوع کلینیک، سن کودک، بهره ی هوشی، تعداد جلسات کاردرمانی و سطح طبقه ای عملکرد حرکتی درشت به صورت تصادفی در دو گروه کنترل و مداخله قرار گرفتند.ابزار جمع آوری داده ها شامل: فرم مشخصات دموگرافیک و آزمون غربالگری تکاملی سنین و مراحل بود. داده ها در 2 نوبت، قبل از مداخله و 3 ماه بعد از مداخله توسط فردی غیر از محقق جمع آوری شد. اجرای مداخله به این شکل بود که محقق در منزل کودکان گروه مداخله حضور می یافت و آموزشهای مربوط به مراقبت های ویژه ی روزمره ی زندگی را به صورت عملی طی 1 جلسه ی 4 ساعته در منزل همراه با تطبیق منزل ارائه می داد. سپس در طول 3 ماه اجرای مداخله را توسط والدین پیگیری می کرد.در نهایت داده ها مورد تجزیه تحلیل آماری قرار گرفتند. یافته‌ها: نتایج نشان داد که پیشرفت سطح تکامل به طور کلی در گروه مداخله نسبت به گروه کنترل بعد از 3 ماه مداخله به طور معناداری بیشتر بود (P=0/015) اما میانگین پیشرفت در ابعاد ارتباط (P=0.713)، حرکات درشت (P=0/2)، حرکات ظریف (P=0/329) و حل مسئله (P=0/981) بین دو گروه معنادار نبود در حالی که میانگین پیشرفت در بعد شخصیتی – اجتماعی تکامل بین دو گروه بعد از 3 ماه مداخله معنادار بود (P<0/001) و این پیشرفت در گروه مداخله بیشتر بود.  نتیجه‌گیری: در این مطالعه اینگونه جمع بندی شد که تطبیق منزل و آموزش مراقبت های ویژه ی روزمره ی زندگی، به مراقبین، در منزل، باعث پیشرفت سطح تکامل به طور کلی در کودکان فلج مغزی 15 تا 60 ماهه می شود.

Title: بررسی روایی و پایایی نسخه فارسی پرسشنامه عادات زندگی (Life-H) درکودکان فلج‌مغزی 13-5 ساله

http://rehabilitationj.uswr.ac.ir/article-1-1432-fa.html

هدف: هدف از این مطالعه، بررسی روایی و پایایی نسخه فارسی پرسشنامه عادات زندگی در کودکان فلج­مغزی 13-5 ساله ایرانی بود. که از آن برای سنجش مشارکت در کودکان فلج­مغزی استفاده می­­گردد. روش بررسی: 101 کودک فلج­مغزی 13-5 ساله و مراقبینشان به روش نمونه­گیری در دسترس وارد این مطالعه که از نوع اعتبارسنجی و ابزارسازی می­باشد؛ شدند. آنها علاوه بر پرسشنامه LIFE-H ، به پرسشنامه ویژگی­های فردی نیز پاسخ دادند. سپس روایی افتراقی آیتم­ها با استفاده از آزمون اسپیرمن محاسبه گردید. برای بررسی پایایی در دفعات آزمون، 22 نفر از آنها به فاصله زمانی 2 هفته، مجدداً پرسشنامه را تکمیل کردند. همچنین روایی ظاهری پرسشنامه در دو مرحله توسط کارشناسان و مراقبین کودکان مورد بررسی قرار گرفت. بررسی روایی محتوایی به روش لاوشه انجام گردید و از نظرات 13 متخصص کاردرمانی استفاده شد. یافته­‌ها: اغلب افراد روایی ظاهری پرسشنامه را خوب گزارش کردند. در بررسی روایی محتوایی به جز دو آیتم، بقیه آیتم­ها نمره قابل قبولی را بدست آوردند. تمام آیتم­ها همبستگی معنادار و خوبی را با نمره بخش مربوطه کسب کردند (r>0/39). همچنین تمام بخش­ها نیز با نمره کل پرسشنامه همبستگی بالاتری نشان دادند (r>0/50). مقادیر ICC برای تمام بخش­ها بیشتر از 0/6 حاصل شد که برای کل پرسشنامه 0/87 بدست آمد. نتیجه‌­گیری: بررسی یافته­‌ها نشان داد که نسخه فارسی پرسشنامه عادات زندگی (LIFE_H) در کودکان فلج­مغزی از روایی و پایایی خوبی برخوردار است. بنابراین می­توان از آن جهت سنجش مشارکت این کودکان هم در مطالعات تحقیقاتی و هم به عنوان ابزار بالینی استفاده کرد.

Title: تأثیر درمان زودرس با داروی لیسکانتین یا پریمیدون بر کاهش تاخیر تکاملی کودکان مبتلا‌به سندرم Drowet و GEFS+

http://rehabilitationj.uswr.ac.ir/article-1-1441-fa.html

هدف: هدف اصلی این مطالعه بررسی تاثیر داروی لیسکانتین یا پریمیدون بر روی کودکان مبتلا‌به سندرم Drowet و GEFS+ می‌باشد. روش بررسی: این مطالعه از نوع کارآزمایی بالینی بوده و در بین کودکان دارای سندرم Drowet و GEFS+ مراجعه‌کننده‌به مرکز طبی کودکان در سال ۱۳۹۱ انجام گرفته است. ۸۶ کودک با تشخیص تب و تشنج وارد مطالعه شدند که ۶ نفر از آن‌ها با توجه‌به تکرار تشنجات و همچنین وجود فوکالیته در هنگام تشنج مبتلا‌به سندرم Drowet و GEFS+ تشخیص داده شدند و در طول یک سال با داروی لیسکانتین یا پریمیدون با دوز ۲۰تا۴۰میلی‌گرم بر کیلوگرم تحت درمان قرار گرفتند. به‌منظور تجزیه و تحلیل داده‌ها از آزمون‌های آماری T-test و Chi Square استفاده شد. یافته‌ها: در طول یک سال بررسی نمونه‌ها ۵ نفر از کودکان به‌دلیل کاهش میزان تشنجات و شروع به‌موقع و دوز مناسب دارو تاخیر یا پسرفت تکاملی از خود بروز ندادند و تنها یک بیمار به‌دلیل تکرار تشنجات و عدم پاسخ مناسب‌به درمان دچار تاخیر تکاملی گردید. نتیجه‌گیری: شروع زودرس درمان داروئی با لیسکانتین یا پریمیدون در کودکان مبتلا‌به تب وتشنج تکرارشونده در پیشگیری از اختلالات تکاملی تاثیر بالائی دارد.

Title: تأثیر آموزش هوش هیجانی بر کاهش پرخاشگری و بیش فعالی دانش آموزان دختر کم توان جسمی-حرکتی

http://rehabilitationj.uswr.ac.ir/article-1-1397-fa.html

هدف: هوش هیجانی دارای نقش حیاتی در سلامت روانی و اجتماعی کودکان می باشد و نقص آن به معنای آماده بودن زمینه وقوع انواع مشکلات و مسائل در کودکان خواهد بود .این مطالعه با هدف اثر بخشی آموزش هوش هیجانی بر کاهش پرخاشگری و بیش فعالی در دانش آموزان دختر کم توان جسمی – حرکتی صورت گرفت. روش بررسی: این مطالعه از نوع تجربی با پیش آزمون پس آزمون با گروه کنترل می باشد. نمونه ی مورد مطالعه 40 دختر دارای کم توانی جسمی–حرکتی کلاس سوم تا پنجم ابتدایی بودند که از طریق نمونه گیری در دسترس انتخاب گردیدند (20 نفر در گروه آزمایش و 20 نفر در گروه کنترل ). ابزار جمع آوری داده ها ،پرسشنامه راتر (فرم مربی، فرم والدین) بود . از روش آماری تحلیل کواریانس چند متغییری برای تجزیه و تحلیل داده ها استفاده شد و تحلیل آماری توسط نرم افزار spss انجام پذیرفت. یافته‌ها: بین هوش هیجانی دانش آموزان کم توان جسمی – حرکتی وپرخاشگری و بیش فعالی آنها رابطه منفی معنادار مشاهده شد. (P<0/001 ,f=21/25) آموزش هوش هیجانی بر پرخاشگری و بیش فعالی دانش آموزان دختر کم توان جسمی-حرکتی تاثیر داشت و منجر به کاهش آن شد( P<0/001 ,f=15/04). نتیجه‌گیری: از آموزش هوش هیجانی می توان جهت کاهش مشکلات رفتاری در دانش آموزان کم توان جسمی -حرکتی استفاده کرد.
